# Supplementary material for: Iterative improvement in the automatic modular design of robot swarms
Source: PeerJ Comput Sci. 2020 Dec 7;6:e322. doi: 10.7717/peerj-cs.322 (PMC7924708; doi:10.7717/peerj-cs.322)
Supplement: Supplemental Information 3 [file peerj-cs-06-322-s003.zip › argos3/doc/api/standalone/a00403_source.html]

ARGoS: core/utility/profiler/profiler.h Source File


- Main Page
- Related Pages
- Namespaces
- Classes
- Files

- File List
- File Members

# core/utility/profiler/profiler.h

Go to the documentation of this file.

```
00001 
00006 #ifndef PROFILER_H
00007 #define PROFILER_H
00008 
00009 #include <sys/time.h>
00010 #include <sys/resource.h>
00011 #include <pthread.h>
00012 
00013 #include <string>
00014 #include <iostream>
00015 #include <fstream>
00016 #include <vector>
00017 
00018 namespace argos {
00019 
00020    class CProfiler {
00021 
00022    public:
00023 
00024       CProfiler(const std::string& str_file_name,
00025                 bool b_trunc=true);
00026       ~CProfiler();
00027 
00028       void Start();
00029       void Stop();
00030       void Flush(bool b_human_readable);
00031       void CollectThreadResourceUsage();
00032 
00033    private:
00034 
00035       void StartWallClock();
00036       void StopWallClock();
00037 
00038       void StartCPUProfiler();
00039       void StopCPUProfiler();
00040 
00041       void FlushHumanReadable();
00042       void FlushAsTable();
00043 
00044    private:
00045 
00046       std::ofstream m_cOutFile;
00047       ::timeval m_tWallClockStart;
00048       ::timeval m_tWallClockEnd;
00049       ::rusage m_tResourceUsageStart;
00050       ::rusage m_tResourceUsageEnd;
00051       std::vector< ::rusage > m_vecThreadResourceUsage;
00052       pthread_mutex_t m_tThreadResourceUsageMutex;
00053 
00054    };
00055 
00056 }
00057 
00058 #endif
```

---

Generated on 10 Jul 2018 for ARGoS by 
 1.6.1
